# Serotonin induces or inhibits neuritic regeneration of leech CNS neurons depending on neuronal identity

**DOI:** 10.1590/1414-431X20187988

**Published:** 2019-02-14

**Authors:** J. Vargas, A. Alfaro-Rodríguez, J. Perez-Orive

**Affiliations:** 1 Regeneration Laboratory, National Rehabilitation Institute “Luis Guillermo Ibarra Ibarra”, Col. Arenal de Guadalupe, Delegacion Tlalpan, Mexico City, Mexico; 2 Neuroscience Division, National Rehabilitation Institute “Luis Guillermo Ibarra Ibarra”, Col. Arenal de Guadalupe, Delegacion Tlalpan, Mexico City, Mexico

**Keywords:** Neuronal regeneration, Serotonin, Leech, Neuronal culture, Pharmacological treatment

## Abstract

Recovery of motor function after central nervous system (CNS) injury is dependent on the regeneration capacity of the nervous system, which is a multifactorial process influenced, among other things, by the role of neuromodulators such as serotonin. The neurotransmitter serotonin can promote neuronal regeneration but there are also reports of it causing restriction, so it is important to clarify these divergent findings in order to understand the direct scope and side effects of potential pharmacological treatments. We evaluated the effect of serotonin on the extent of neuritic outgrowth and morphology of three different neuronal types in the leech *Haementeria officinalis* during their regeneration *in vitro:* Retzius interneurons (Rz), annulus erector (AE) motoneurons, and anterolateral number 1 (AL1) CNS neurons. Neurons were isolated and cultured in L15 medium, with or without serotonin. Growth parameters were registered and quantified, and observed differences were analyzed. The addition of serotonin was found to induce AL1 neurons to increase their average growth dramatically by 8.3-fold (P=0.02; n=5), and to have no clear effect on AE motoneurons (P=0.44; n=5). For Rz interneurons, which normally do not regenerate their neurites, the addition of concanavaline-A causes substantial growth, which serotonin was found to inhibit on average by 98% (P=0.02; n=5). The number of primary neurites and their branches were also affected. These results reveal that depending on the neuronal type, serotonin can promote, inhibit, or have no effect on neuronal regeneration. This suggests that after CNS injury, non-specific pharmacological treatments affecting serotonin may have different effects on different neuronal populations.

## Introduction

After central nervous system (CNS) injury, reestablishing motor behaviors and cognitive function depends on neuronal regeneration and outgrowth of nerve fibers that bypass the injury and correctly connect with their targets. Failure of this process can lead to various motor and cognitive deficits in higher vertebrates. Although there have been advances in the fields of neuronal regeneration and motor rehabilitation, available therapies tend to focus on strengthening intact connections and there is yet no totally effective treatment available. Recovery of motor function is still dependent on the nervous system's regeneration capacity.

Neuronal regeneration is a multifactorial phenomenon, dependent on the injured neurons' survival, interaction with other neurons, soluble factors, neuronal activity, and extracellular matrix proteins, among other factors. Neurotransmitters and their receptors are also key factors of neuronal regeneration. These can be secreted and expressed during development when neuronal circuits are beginning to form, suggesting that neurotransmitters such as serotonin may play important roles in these processes. For example, in addition to regulating many behaviors and neuropsychological processes (1–4), serotonin has been shown to participate in guiding growth cones in culture, as well as in axonal extension and synaptogenesis (5,6). These effects have been shown in vertebrates and invertebrates (7–12). However, the addition of serotonin to culture medium has also been demonstrated to inhibit neuritic outgrowth (9 [Bibr B10],^11^). Typically, neurotransmitters diffuse in the synaptic cleft, at relatively short distances from their secretion site; however, somatic secretion of serotonin has been reported (12–14), allowing it to have an effect on processes, such as axonal regeneration, that occur outside of synapses. Also, serotonin regulates neuronal excitability by activating or inactivating various ion channels, and it can also regulate the electric response of neuronal groups by synchronizing their activity (15). This confers to serotonin the potential to regulate neuritic extension in neuronal populations, which may represent a temporal window determined during development or in the adult (16,17).

The fact that serotonin induces neurite outgrowth in some neurons but retraction in others poses the challenge to understand what determines the effect that this neuromodulator has on neuronal regeneration.

The complexity of the CNS in higher vertebrates renders its study challenging and therefore, many key principles of neural functioning have been first explored and uncovered in simpler invertebrate systems (18–20). One invertebrate nervous system that has been investigated intensively is the leech (21,22). This animal's CNS regenerates neural connections after injury and it regains mobility in a few days. The neurons in the leech's CNS can be identified and isolated very precisely and when cultured, they regenerate characteristic morphological patterns, dependent on their identity and substrate. This study explores the differential effect that serotonin has on neuronal regeneration by analyzing in culture its role in the neuritic outgrowth of Retzius (Rz) serotonergic interneurons, annulus erector (AE) motoneurons, and anterolateral number 1 (AL1) neurons in the leech *Haementeria officinalis*. AL1 neurons are similar to the anterior pagoda neurons of the medicinal leech *Hirudomedicinalis,* in terms of their localization in the ganglion and their morphologic regeneration patterns. Quantitative analysis included the measurement of the total neuritic length, the number of primary neurites, and the number of ramifications.

## Material and Methods

### Central nervous system of *Haementeria officinalis*

**Figure 1.**
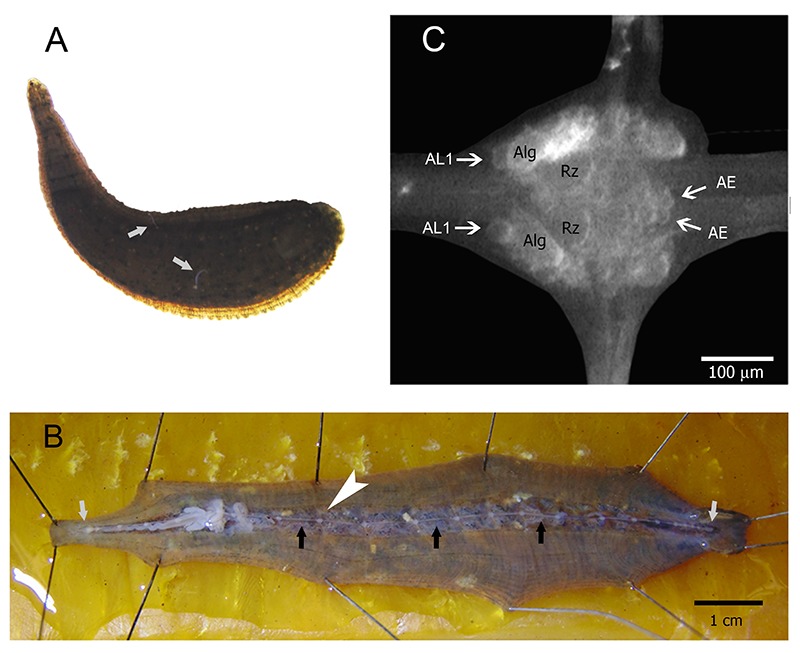
Central nervous system (CNS) of the Mexican leech *Haementeria officinalis*. **A**, Dorsal view of an experimental animal. The head is on the left superior corner of the image and the tail is at the other end. It has 2 ventral suckers at both ends. The image shows two spermatophores (sperm bags, white arrows) deposited during fertilization. **B**, The animal was anesthetized and pinned on wax. The CNS, consisting of a ganglion chain, was visualized after ventral incision. The chain extends from the head (left end) to the tail and includes 4 ganglia in the head and 7 in the tail (white arrows). The 21 intermediate ganglia are linked by connective nerves; one ganglion is shown with a white arrowhead and three connective nerves are shown with black arrows. **C**, Intermediate ganglion. The position of the neurons is stereotyped. The Retzius (Rz) interneurons are in the ventral area, the annulus erector (AE) motoneurons are posterior and the anterolateral number 1 (AL1) neurons are located anteriorly to the giant anterolateral neurons (Alg).

Experiments were conducted on Mexican leeches (*Haementeria officinalis*, Figure 1A), initially collected in lakes and dams in Mexico's central plateau by aprovider licensed by the local "Dirección de Desarrollo Agropecuario" and then grown and reproduced in a colony in the laboratory as described below. This organism belongs to the Phylum Annelida, Class Clitellata, Order Rhynchobdellida and Family Glossiphoniidae. Its CNS consists of a ventral ganglion chain with 21 intermediate ganglia and two cumuli of ganglia fused to the head and tail (Figure 1B). Each ganglion contains approximately 400 neurons (Figure 1C). All ganglia are identical except for the 5th and 6th segmental ganglia, which are associated to the animal's reproductive functions and have a different number of neurons than the rest. The position and function of the neurons in each ganglion are stereotyped. For example, Retzius interneurons are localized in the central area of the ganglion, AE motoneurons in the posterior part, and the AL1 neurons are always associated to the anterior portion of the Alg neurons, the largest neurons in the ganglion.

### Animal maintenance and culture of neurons

Leeches were kept in fish tanks (15 animals per tank) filled with 4 L of potable water (E-pura, Mexico) and a gravel bottom, at a temperature of 23°C, until used. All procedures were approved by the Institutional Committee for Care and Use of Laboratory Animals (INR-CICUAL No. 15/11). To obtain the Rz, AE, and AL1 neurons, adults weighing 0.4 to 0.6 g were anesthetized for 10 min in a 9% ethanol solution. The animal was subsequently pinned ventral side up on a plate with wax. A ventral incision of the entire animal's length allowed the visualization of the connective nerve (Figure 1B). With forceps and microdissection scissors, the ganglion chain was removed and placed in a Petri dish covered in silicon and 3 mL of L15 medium (Sigma, USA) and 2% fetal bovine serum (Gibco, Mexico). The ganglion chain was ventrally fixed with micropins and, with the neurons exposed to the medium, it was incubated for 30 min with the enzyme collagenase dispase (2 mg/mL. Roche, Germany) in order to dissolve the extracellular matrix. The medium was then replaced with fresh medium and the neurons of interest were removed with a glass pipette whose distal end was adjusted to the neuron's diameter. Neurons were washed in 5 drops of 100 µL medium each to sterilize them and eliminate any remnants of extracellular matrix. Finally, the neurons were seeded on the glass-bottom dishes, with the stump firmly in contact with the substrate, since this leads to the genesis of new neurites during the regeneration process. In some records, neurons appear to lack a stump because the soma was on top, hiding its presence.

### Addition of serotonin to the culture

Different serotonin hydrochloride concentrations (5-HT, Sigma) were used (2.35, 23.5, 47, 188, and 377 µM) in order to establish the interval with the greatest effect. Finally, the experiments were performed with 188 µM, since this concentration yielded the clearest effects. The serotonin solution was added to the cultures once the neurons had been seeded.

### Neuron culture in concanavaline A (Con-A)

Con-A, a substrate on which neurons grow extensively, was used to better evaluate the effect of serotonin on Rz cells, which normally have very limited regeneration (see Results). Rz neurons were cultured on glass plates covered with Con-A (Sigma). This lectin (2 mg/mL) was filtered and applied to the glass surface. After sedimentation for 2 h, the culture plate was washed three times with deionized water, and L15 medium was added for the neuronal culture.

### Analyzed parameter recordings

Images of cultured neurons were obtained 48 h after initiation of growth in culture to determine growth with phase contrast microscopy. They were then digitally stored for subsequent quantification of total neuritic length (TNL), number of primary neurites (NPN), and number of total ramifications (NTR). Each experiment consisted of culturing a neuron from each type, cultured with or without serotonin, taken from a single animal. Between 4 and 5 independent experiments were conducted in each condition (with and without serotonin). All three parameters were measured in each neuron. Quantification of neuritic length was performed with the NeuronGrowth program (23), a free online plug-in to the ImageJ program (NIH, USA) for image analysis (24). In the program, we used a minimum value of 7 and a maximum of 10 units in thickness in the windows of the automatic neurite tracer. With these values, neurites measuring less than 3 µm in diameter were excluded from analysis. The Rz neurons cultured with the lectin Con-A, yielded neurites in the shape of extensive lamellipodia and in this case, the total neuritic area (TNA) was quantified only using the ImageJ program.

### Statistical analysis

Analysis of morphological parameters was performed with parametric statistics. Data, including all length measurements, are reported as means±SE. The level of significance was established at the 95% level and calculated with unpaired *t*-tests. Calculations were made using the Sigma Plot 2001 package (Systat Software Inc., USA).

## Results

### Rz interneuron neuritic growth patterns

**Figure 2.**
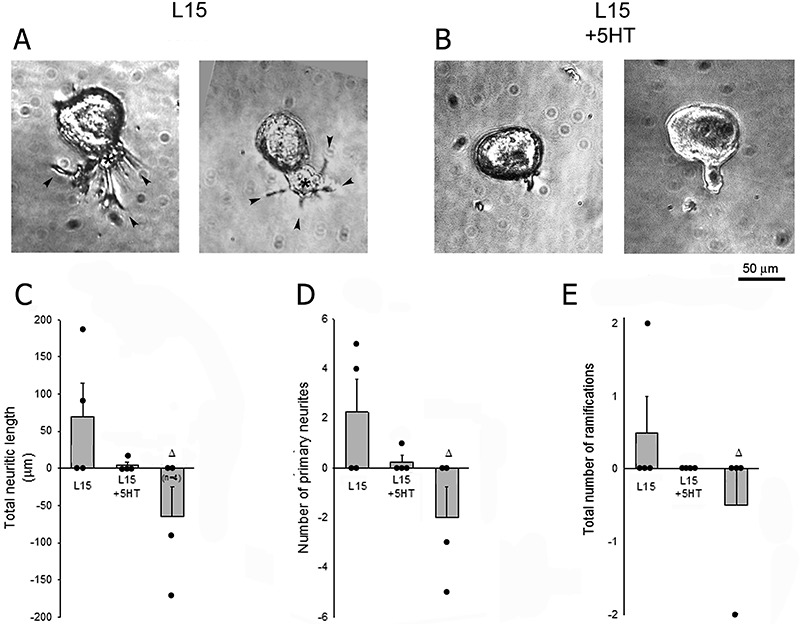
Effect of serotonin (5-HT) on the morphological pattern of Retzius interneurons. **A**, Upon removal, these neurons maintain a robust stump (asterisks) from which neurites are formed. When cultured on glass plates, their growth is scant, with some primary neurites and few ramifications (black arrowheads). **B**, The presence of serotonin decreased neurite extension and many neurons did not yield neurites or these were even shorter. **C**–**E**, In these neurons, serotonin decreased the magnitude of the average total neuritic length, the number of primary neurites, and the number of branches. Δ denotes the average difference (‘with 5HT’ minus ‘without 5HT’) for each pair of neurons from the same experiment. However, given the limited growth occurring in the Retzius neurons under control conditions, these reductions in growth were not statistically significant (unpaired *t*-test). Data are reported as means±SE.

**Figure 3.**
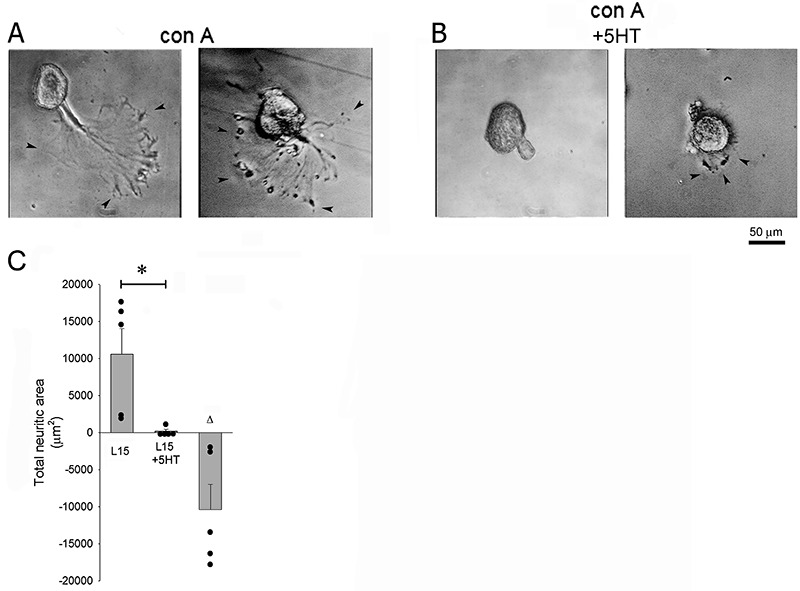
Effect of serotonin (5-HT) on growth of Retzius neurons cultured on the lectin concanavaline-A (Con-A). **A**, Retzius neurons cultured with the lectin Con-A yield neurites in the shape of extensive fan-like lamellipodia. The arrowheads show the edges of these structures. **B**, The presence of serotonin in the medium decreased the lamellipodia's extension, and some neurons only yielded short and thin neurites. **C**, The total growth area decreased by 98%. Δ denotes the average difference (‘with 5HT’ minus ‘without 5HT’) for each pair of neurons from the same experiment. Data are reported as means±SE. The asterisk denotes 95% statistical significance with the unpaired *t*-test.

After 48 h in culture, some Rz serotonergic interneurons seeded on glass plates yielded a few short and minimally ramified neurites (Figure 2A; n=4). These neurons generated on average 2.25±1.31 primary neurites with a total length of 69.53±44.76 µm (Figure 2C–E). The addition of serotonin decreased the average neuritic length 94% to 4.25±4.25 µm (n=4), with 0.25±0.25 primary neurites and no ramifications (Figure 2B–E, Table 1). Due to the scarce growth of these neurons under control conditions (without serotonin), these results, although suggesting an inhibition of growth with serotonin, were not statistically significant (P=0.20 for neurite length, and P=0.19 for number of primary neurites). Thus, to better evaluate the effect of serotonin, this neuronal cell type was cultured with Con-A, a substrate on which neurons grow extensively. On this substrate, these neurons regenerated large lamellipodia with phyllopodia and varicosities on the edges (Figure 3A). In the absence of serotonin, the extension of these lamellipodia was 10,598±3,473 µm^2^ (Figure 3C). The presence of serotonin inhibited the growth of these neurons: lamellipodia were less extensive (219±219 µm^2^, P=0.02, n=5, Figure 3C, Table 1) and also had phyllopodia and varicosities on their edges (Figure 3B).

**Table 1.** Changes in morphological parameters induced by serotonin (5-HT).

	TNL	NPN	TNR	TNA
Rz	69.5±44.8	2.3±1.3	0.5±0.5	10598±3473*
Rz + 5HT	4.3±4.3	0.3±0.3	0.0±0.0	219±219*
Δ	−65.3±41.1	−2.0±1.2	−0.5±0.5	−10379±3418*
AE	83.0±36.7	1.4±0.7	0.4±0.4	
AE + 5HT	49.6±19.4	1.4±0.5	0.4±0.2	
Δ	−33.4±29.6	0.0±0.7	0.0±0.6	
AL1	60.9±30.0*	1.2±0.6	0.4±0.2*	
AL1 + 5HT	504.3±154.3*	3.2±0.9	7.2±2.6*	
Δ	443.4±135.8*	2.0±1.2	6.8±2.4*	

Data are reported as average values and standard error. The asterisks indicate statistically significant differences with the unpaired *t*-test. TNL: total neuritic length (µm); NPN: number of primary neurites; TNR: total number of ramifications; TNA: total neuritic area (µm^2^); Rz: Retzius interneurons; AE: annulus erector motoneurons; AL1: anterolateral number 1 neurons; Δ denotes the average difference (‘with 5HT’ minus ‘without 5HT’) for each pair of neurons from the same experiment.

### AE motoneuron neuritic growth patterns

**Figure 4.**
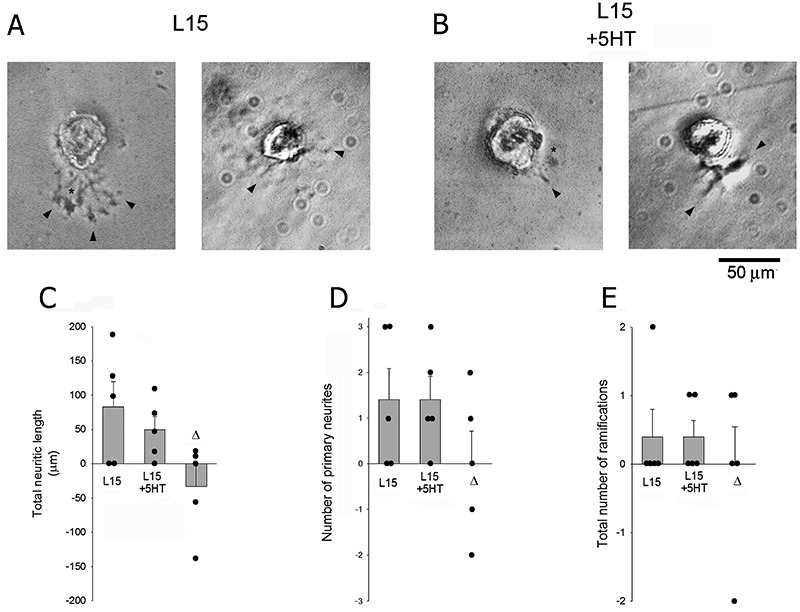
Effect of serotonin (5-HT) on the morphological pattern of annulus erector (AE) motoneurons. **A**, AE motoneurons yielded few short and thick primary neurites (black arrowheads), with some ramifications and lamellipodia (asterisks). **B**–**E**, The presence of serotonin in the medium did not modify the morphological parameters. Δ denotes the average difference (‘with 5HT’ minus ‘without 5HT’) for each pair of neurons from the same experiment. No statistical significance was found with the unpaired *t*-test. Data are reported as means±SE.

After 48 h in culture, AE motoneurons yielded short and broad neurites and some developed small lamellipodia in the proximal region (Figure 4A). Total average neurite length was 82.98±36.71 µm (n=5, Table 1), with 1.40±0.68 primary neurites and 0.40±0.40 ramifications in total (Figure 4C–E). The addition of serotonin did not modify the morphological pattern, the neurons regenerated thick neurites from the stump, and in some cases, there was also lamellipodia formation. The neurons also developed scant ramifications (Figure 4B). In these neurons, serotonin did not cause a significant change in any of the morphological parameters explored: average neuritic length was 49.59±19.42 µm (P=0.44, n=5), with the same averages of 1.40±0.51 for primary neurites and for 0.40±0.24 total ramifications (Figure 4C–E, Table 1).

### AL1 neurons neuritic growth patterns

**Figure 5.**
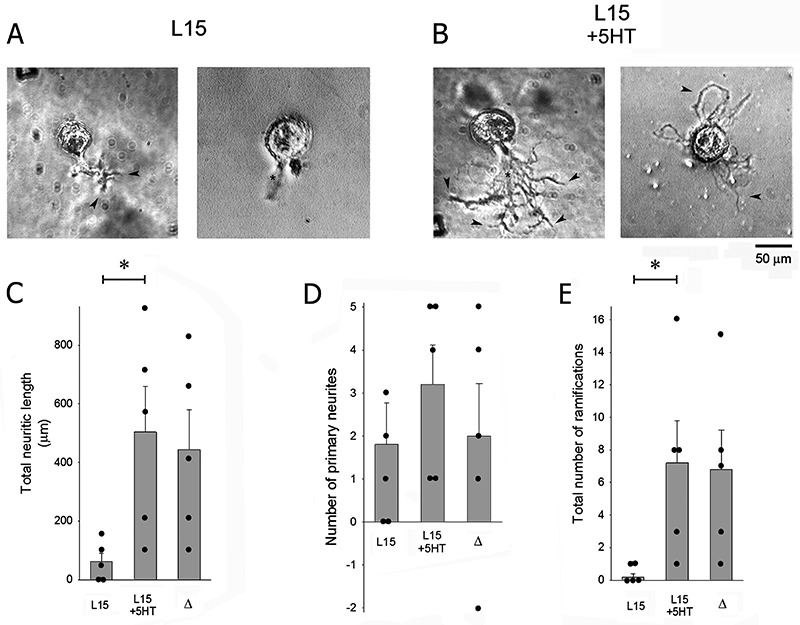
Effect of serotonin (5HT) on the morphologic pattern of AL1 neurons. **A**, Upon removal and culture on glass plates, they yield short primary neurites with few ramifications. The arrowheads show several neurites and the asterisks show some lamellipodia. **B**, The presence of serotonin induced extensive neurite growth and increased its ramifications but had no effect on the number of primary neurites. **C**-**E**, The neurotransmitter increased the total neuritic length 8.3-fold and the ramifications18-fold, but had a smaller (non-statistically significant) effect on the number of primary neurites. Δ denotes the average difference (‘with 5HT’ minus ‘without 5HT’) for each pair of neurons from the same experiment. Asterisks denote 95% statistical significance with the unpaired *t*-test. Data are reported as means±SE.

In the absence of serotonin, AL1 neuron neuritic growth was also scarce and with few ramifications (Figure 5A). Average neuritic length was 60.85±30.03 µm (n=5, Table 1), with 1.20±0.58 primary neurites and 0.40±0.24 total ramifications (Figure 5C–E). Adding serotonin increased the average neurite length and neurites developed a curved and a much ramified aspect (Figure 5B). Average neuritic length increased 8.3-fold to 504.28±154.27 µm (P=0.02, n=5) with 3.20±0.92 primary neurites (P=0.10) and an increase in total branching to 7.20±2.60 (p=0.03) (Figure 5C–E, Table 1).

## Discussion

**Figure 6.**
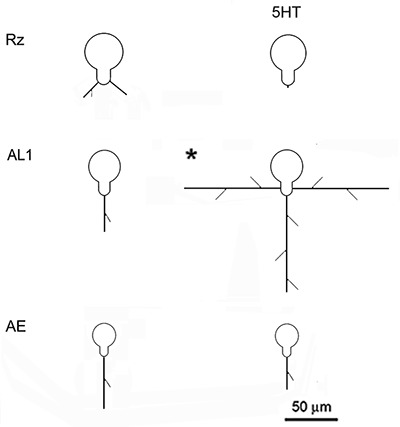
Morphological model of the growth of Retzius (Rz), anterolateral number 1 (AL1) and annulus erector (AE) neurons. Neurite sizes are drawn to scale based on average results. After adding serotonin (5-HT) to the medium, the total neuritic length and the number of primary neurites and ramifications were decreased in the Rz neurons. In AL1 neurons, total neuritic length (TNL) and number of ramifications increased. There was no clear effect on AE motoneurons. The asterisks indicate 95% statistically significant differences in TNL with the unpaired *t*-test.

This study showed that serotonin promoted the neuritic extension of AL1 neurons, inhibited that of Rz interneurons, and had no effect on AE motoneurons. Serotonin also was found to induce changes in the number of neurites and ramifications. These effects are shown in the morphological model of growth under the effect of serotonin (Figure 6). The presence of this neurotransmitter induced an increase in all parameters in the AL1 neurons. The average neuritic length increased 8.3-fold, the number of primary neurites 2.7-fold, and the ramifications 18-fold. The effect on the morphology of the Rz interneurons was opposite that of the AL1 neurons. In the Rz interneurons, the average neuritic length, number of primary neurites, and ramifications were all decreased in the presence of serotonin. Due to the very limited amount of regeneration in these neurons in the control condition, these further reductions, although all directionally similar, were not statistically significant. Adding Con-A, a substrate in which neurons grow extensively, allowed for a better characterization of the effect of serotonin on Rz interneurons. Under these conditions, the addition of serotonin was found to decrease the extent of the lamellipodia by 98% (and this decrease was found to be statistically significant). However, serotonin had no significant effect on the morphological patterns of the AE motoneurons.

This effect may be mediated by the nature of the receptor activated by serotonin. The three neuronal cell types studied may have different serotonin receptors or lack them altogether, which for example could be the case of the AE motoneurons. The 5HT-1A receptor has been identified in the CNS of *H. medicinalis* by reverse transcription-polymerase chain reaction (RT-PCR) (unpublished data). This receptor has been associated with neuritogenesis inhibition, and in 5HT-1A^-/-^mice, the 1A receptor restricts dendritic growth in hippocampal neurons (25). It would be interesting if future studies determine whether Rz neurons express this receptor isoform, which could potentially be the determining factor of serotonin inhibition in this neuronal type.

Growth induction in AL1 neurons may be mediated by receptors promoting neuronal electric activity, such as types 5HT-2 and 5HT-3. The type 2 receptor has a depolarizing effect on the cell membrane by decreasing the conductance of the potassium inward rectifier (KIR). Depolarization can activate voltage-dependent calcium channels, which in turn may trigger signaling for cytoskeletal polymerization leading to neurite extension (26). Calcium entry is also known to be part of the mechanism through which type 3 receptors mediate neuritic growth (12).

In some instances, we have observed in our laboratory that microglia can sometimes be attached to the somas in culture, and that they can influence the growth of neuritic processes. Microglia are known to possess various types of receptors for serotonin (27), and these cells have been reported to secrete neurotrophic factors promoting neuritogenesis (28). Therefore, it would be interesting if future studies further explored the effect that microglia can have on differential regeneration.

In AL1 neurons, serotonin increased the rate of ramification 18-fold, with a substantially smaller effect of 2.7-fold on the number of primary neurites. This suggests that both mechanisms are separate cellular processes. The formation and distribution of primary neurites relates to cellular polarity, in which RacGTPases (29) play a significant role, whereas the formation of ramifications yielding secondary neurites is mediated by RalGTPases via Gap-43 proteins that are associated to neuronal growth and regeneration (30).

This study showed that, depending on neuronal type, serotonin can inhibit, induce, or have no effect on neuronal regeneration: it exerts an effect on neuritic extension, on the number of primary neurites, and on the ramification rate. This furthers the understanding that serotonin's effect is dependent on neuronal identity and that non-specific pharmacological treatment involving this neurotransmitter may affect several morphological parameters differently in various neuronal populations.
